# Key factors of the willingness of rural populations settling in cities (RPSC) from a Lacanian psychoanalysis theory perspective

**DOI:** 10.1371/journal.pone.0243775

**Published:** 2020-12-22

**Authors:** Zhiheng Yang, Nengneng Shen, Chenxi Li

**Affiliations:** 1 Institute of Regional Economics, Shandong University of Finance and Economics, Jinan, Shandong, China; 2 School of Economics, Shandong University of Finance and Economics, Jinan, Shandong, China; 3 School of Public Administration, Xi’an University of Architecture and Technology, Xi’an, Shaanxi, China; Northeastern University (Shenyang China), CHINA

## Abstract

The migration of populations from rural to urban areas is a typical phenomenon of urbanization in developing countries. Based on Lacanian psychoanalysis theory, this study analyzes the decision-making mechanism of the willingness of rural populations settling in cities (RPSC), and analyzes the key factors that affect the willingness of RPSC by using the binary Logit regression method based on survey data in Changyi, China. The results show that the willingness of RPSC is a realistic choice under the joint action of the ‘mirrored’ incarnation and the ‘non-mirrored’ order. Among the factors, ‘age’, ‘ethnic groups’, ‘educational attainment’, and ‘social intercourse’, representing the ‘mirrored’ incarnation, and ‘communities’ safety gap’, ‘healthcare services policy’, ‘public housing policy’ and ‘employment insurance gap’, representing the ‘non-mirrored’ order, are significant in affecting the willingness of RPSC. These findings validate the adaptability of psychoanalysis to analyze the willingness of RPSC, and increases the understanding of individual willingness and behavioral choice in the context of a specific social background, which can provide decision-making reference for urban and rural planning and public policy makers.

## Introduction

Population migration is an important phenomenon in the development of human society, especially in developing countries. The migration of populations is closely linked to the transformation of the economic and social structure in these countries, which is mainly reflected in rural-to-urban population migration driven by the promotion of urbanization, that is, rural populations settling in cities (RPSC) [1–4]. In China, which is one of the most populous countries in the world, the phenomenon of RPSC is particularly typical. Household registration (‘hukou’) is used to divide the population of China into two categories: rural and urban households. The RPSC refers to the permanent settlement of the rural population in cities, including the full integration of the rural population into the urban economic and sociocultural environment, also known as ‘citizenization’ [5, 6]. From 1978 to 2018, the urban registered population increased from 172 million to 831 million, while the rural registered population decreased from 790 million to 577 million [7]. A large rural registered population moved and settled in cities, improving the income and living standard of the rural population. RPSC is the most profound aspect of the process of urbanization and plays a leading role in promoting the prosperity of urban and rural economies and the overall progress of society [8–11].

Currently, China's urbanization rate based on the resident population living in urban areas has reached 59.58%, which means that China's urbanization has entered a mature stage. However, the urbanization rate calculated by the urban registered population is only 43.37% [7]. The rural population makes a living in the city without urban household registration and is unable to receive treatment equal to the urban registered population. The rural population flows between cities and the countryside and even moves from one city to another to seek new employment opportunities. The phenomenon of rural population floating in cities is the embodiment of the low-cost urban development mode. Rural populations without urban household registration often encounter discrimination in employment, a lack of social welfare, and inability to participate in urban affairs and have no sense of belonging in cities [12]. The rural population often falls into the dilemma of ‘staying in the city’ or ‘going home’. Because this development mode is not conducive to the long-term development of urbanization, a new type of urbanization emerged that proposes the development of a ‘people-oriented’ concept. The new type of urbanization focuses on encouraging rural populations to settle in cities so that they can achieve a better life. China's urbanization rate is still lower than that of the United Kingdom, Japan and other countries with similar population densities. The environmental capacity of existing cities has not yet reached its maximum, and the focus of urbanization strategy is still on increasing the urbanization rate by further promoting ‘citizenization’ based on RPSC [13–15]. The new type of urbanization encourages the development of small and medium-sized cities and optimizes the spatial distribution of cities by encouraging rural populations to settle in small and medium-sized cities. Especially in the face of the COVID-19 outbreak this year, it is time to profoundly rethink the low-cost urbanization development path, encourage the development of small and medium-sized cities and optimizes the spatial distribution of cities by encouraging rural populations to settle in small and medium-sized cities.

In fact, there are abundant theoretical research results to explain rural-urban population migration. For example, population geography proposes the ‘push-pull’ theory, which holds that population migration is the result of the joint action of the push forces of emigration and the pull forces of immigration [16]. From the perspective of the behavior decision-making process of individual migrants, the ‘push-pull’ theory includes two basic hypotheses: one assumes that people's migration behavior is a rational choice, while the other suggests that migrants have a relatively full understanding of information on the original residence and the destination. Only in this way can sound migration choices be made.

Neoclassical economists introduce the concepts of economics and supply and demand into the study of rural-urban population migration. They believe that regional differences in the demand and supply of labor cause the adjustment of labor between different regions, and rural-urban population migration is the embodiment of this adjustment process. The American development economist Todaro [17], starting from the urban labor market, believes that the willingness of RPSC could be explained by the ‘expected returns’. Because cities provide more employment opportunities and higher incomes, large rural populations migrate to cities and towns. The theoretical premise of these studies is the assumption that the research object is the ‘rational man’ and that the migrating population will provide ideal feedback on policy regulation.

However, in practice, the migration of rural populations to cities does not occur in line with the theoretical assumption. The reason is the differences in individual understanding of the optimal choice and decision-making environment. There is a trend toward using the ‘behavioral man’ to replace the ‘rational man’ in economics, focusing on the influence of social motivation on individual decision-making [18]. Simon [19, 20] demonstrates that the individual psychological process is the factor that restricts rational maximum decision-making, and Kahneman and Tversky [21] propose the prospect theory for decision-making behavior in uncertain situations, which started research on the influence of various behaviors and the factors that affect behavior in individual decision-making. Psychology has received considerable attention due to its exploration of individual decision-making mechanisms [22]. It is used to test the effect of policy related to individual decision-making behavior in practice and provides a useful reference for similar research and policy practice [23].

RPSC is a complex social system project that represents the transformation of the rural population in terms of social identity, occupation, role consciousness, ideology and social rights and reflects the city's acceptance of the rural population. There are abundant researches in the field of rural population's willingness to settle in the city, mainly focusing on the extraction of the dominant factors influencing the willingness, like economic, social, cultural, psychological and other fields [24–27]. These studies show the diversity and hierarchy of factors affecting RPSC. However, they focus primarily on the expression of the correlation between the influencing factors and RPSC willingness based on the enumeration method, and the mechanism of the internal relations among the factors is not clear. Previous studies pay less attention to the factors that generate the willingness of RPSC with regard to the behavior decision-making process, and less focus on the migration and settlement of rural populations in small and medium-sized cities.

In this study, we introduce the concept and method of psychoanalysis to analyze the rational logic of the formation of RPSC willingness in small and medium-sized cities, explore the decision-making mechanism of the willingness of RPSC, analyze the influencing factors of the willingness of RPSC in Changyi, and provide a useful reference for decision makers of RPSC to better design and choose policies. The objectives of this study are to (a) address the mechanism of the formation of RPSC willingness from the perspective of Lacanian psychoanalytic theory and (b) conduct an empirical study to demonstrate the research hypotheses and identify the key influencing factors that affect the willingness of RPSC.

The main contributions of this paper are as follows: (1) identifying the Willingness of rural populations settling in cities (RPSC) based on Lacanian Psychoanalysis; (2) exploring the mechanism of the generating and changing process of the willingness of RPSC in small and medium-sized cities; (3) providing a factual basis for local government to formulate the development strategy of promoting ‘citizenization’ and deepening urbanization.

## Theoretical analysis

### Lacanian psychoanalytic theory

To prevent the ‘eternity’ pursued by mankind from being built on a weak material foundation, philosophy has long been the foundation of the development of human society. Interpreting the objective world from the spiritual level has been a feasible way of solving practical problems. Since industrialization, psychology has developed as an independent discipline. Lacanian psychoanalytic theory, as one of the branches of post-Freudian psychoanalytic theory, has opened up the post-structural ideological system of psychoanalysis. Lacanian psychoanalysis re-explains the ‘subject’, ‘unconscious’, ‘other’, ‘desire’, and other core psychological concepts. The ‘subject’ is ‘unconscious’, usually reduced to subjection. The ‘unconscious’ is the discourse of the ‘Other’, which is inserted into the symbolic order like a language structure from the outside and operates according to different relations in the symbolic order.

In Lacan’s terms, ‘All sorts of things in this world behave like mirrors’ [28]. In infancy, the subject recognizes itself through the ‘mirror’, namely, the Other, to generate desire. The subject's desire is the desire of the Other, which is not the actual object of desire but rather the object cause of desire [29]. In this sense, the Other is an expectation. Johnston [30] states that Lacanian psychoanalytic theory symbolizes desire as ‘mirrored’ (i.e., incarnated in visible avatars) and ‘non-mirrored’ (i.e., incapable of being captured in spatiotemporal representations). Especially for people in a certain process of social development, the formation of consciousness and the occurrence of behavior are at the same time. In the group, the unconscious behavior of all members has little difference, that is, the unconscious behavior has consistency. The ‘mirrored’ is therefore not a real other person but an imagination of oneself, an object that one desires to be; the ‘non-mirrored’ is a symbolic order, including laws and regulations. The ‘mirrored’ incarnation and the ‘non-mirrored’ order constitute the desire logic by which the Other regulates unconscious subjects.

Lacanian psychoanalytic theory provides an analytical framework for understanding the ideology of the population. Some scholars have attempted to introduce this analytical perspective into the fields of human geography and urban and rural planning. For example, Nichols [31] focuses on the emotional dimension of human mobility and combines Lacanian psychoanalysis and landscape analysis to interpret unconscious urban expressions. Yang [32] analyzes the situation of foreign talent entering Singapore from the perspective of Lacanian psychoanalysis and attempts to explain their migration and mobility. However, few correlative studies have been reported, and this analytical perspective has not yet been applied to the field of the willingness of RPSC.

### The framework of the willingness of RPSC based on Lacanian psychoanalysis

As to the psychological mechanism of the willingness of RPSC, unconscious subjects are all blind in a wide sociocultural context, rural populations have no desire to settle in cities without the influence of urbanization. Due to the urban-rural development gap, the prosperous urban lifestyle is taken as a mirror faced by the rural population [33]. Through the mirror, the rural population clearly recognizes the backwardness and inadequacy of their living environment, which is regarded as the Other. Inspired by the Other, the desire of the rural population is aroused to settle in the city, that is, the willingness of RPSC.

Driven by desire, the rural population begins to pour into cities. The first thing they face is the need to find jobs to survive. Therefore, the rural population must accept the selection of the urban labor market. This state can be abstracted as the mode of ‘looking in the mirror’. The reality is that only part of the rural population can be accepted by the urban labor market. The individual characteristics of the selected rural population can be integrated into an image, which can be called the ‘mirrored’ incarnation of a successful rural population. By ‘looking in the mirror’, the rural populations that do not match the ‘mirrored’ incarnation will actively reduce their willingness of RPSC.

In the process of ‘looking in the mirror’, the rural population finds that what they see is only part of the prosperous urban lifestyle. In different cities, there are large or small mirrors provided to the rural population. The size of the mirror determines the vision that the subject can see because it is limited by the mirror frame. The mirror frame can be regarded as the external institutional environment, which is determined by urban public policies and rules, including various social insurance benefits and public welfare services related to cities; this can be called the ‘non-mirrored’ order. The ‘non-mirrored’ order represents the urban acceptance of the rural population, which has no choice but to abide by the ‘non-mirrored’ order. In reality, the social welfare division of urban and rural household registration systems excludes rural populations. The limitations of these realities make rural populations seriously consider whether they will settle in cities.

**Fig 1:**
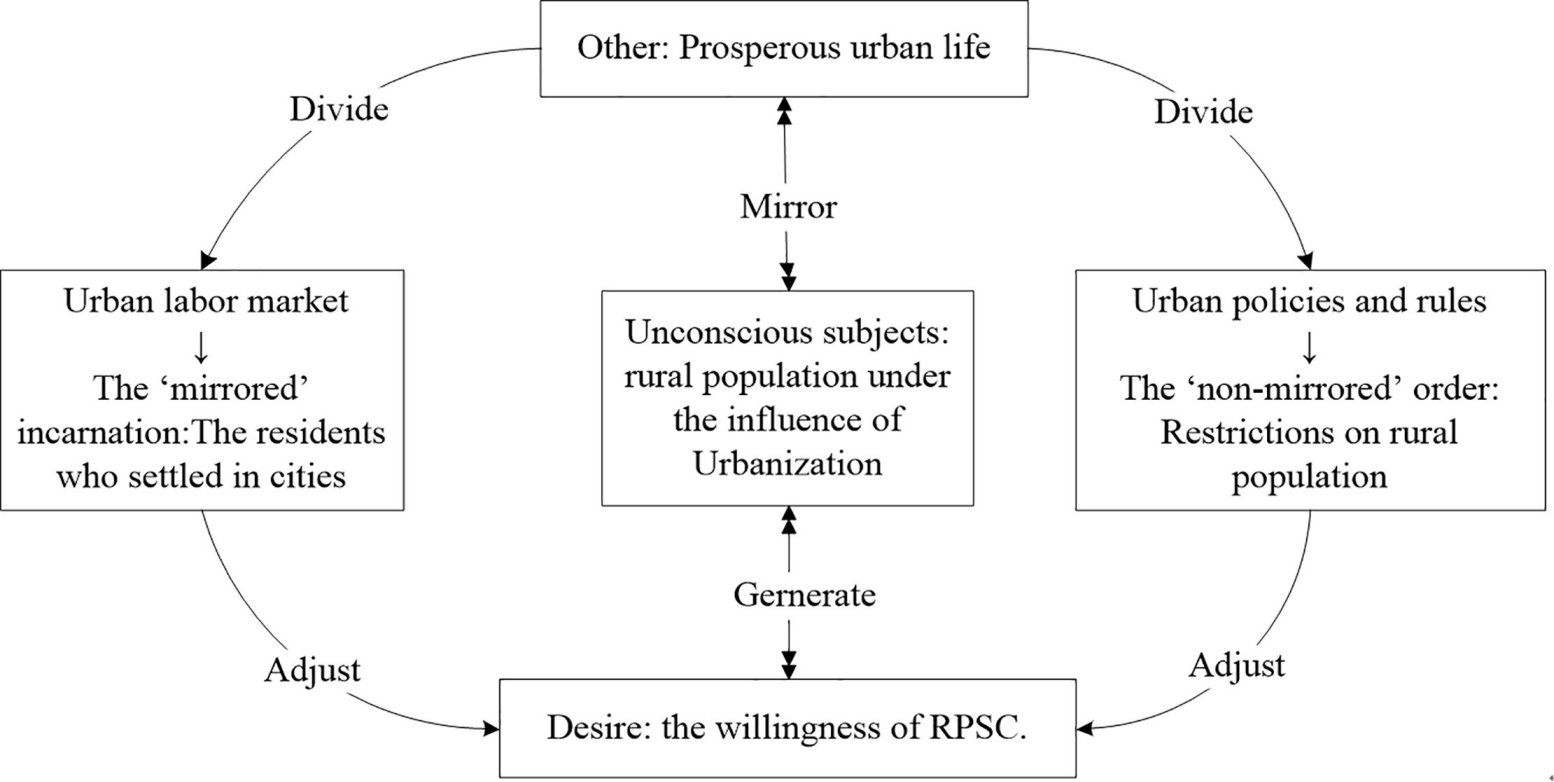
Theoretical analysis framework.

Finally, under the joint effect of urban labor market selection and urban social insurance and public welfare, the desire of the rural population is constantly stimulated in reality. The willingness of RPSC is the partial expression of desire under realistic conditions and is the interaction between the subject and the Other (see Fig 1).

This confirms Lacan's view that: the unconscious does not ‘belong’ to the individual and is an effect of signification on the subject. The emergence of consciousness is influenced by individual innate endowment, individual behavior, family environment, community environment, living conditions, working conditions, economy, culture, health and environment of the city where they live and relevant policies, which correspond to the ‘mirrored’ incarnation and the ‘non-mirrored’ order’ variables, respectively. The ‘mirrored’ incarnation represents a stage of psychological change: the rural population passively adapts to the selection of the urban labor market and actively adjusts personal willingness. That is, the selection of the urban labor market causes a change in RPSC willingness. The ‘non-mirrored’ order represents another stage of psychological change: according to the availability of urban social insurance and public welfare, the rural population adjusts their willingness to settle in the city. In the face of the selection of the urban labor market and the restriction of urban social insurance and public welfare, the rural population evaluates their own capabilities and specific interests, weighs and decides whether to settle down, and finally forms their actual will.

Based on the qualitative analysis of the decision-making mechanism of RPSC willingness formation, this study proposes the following research hypothesis: the willingness of RPSC is a realistic choice under the joint action of the ‘mirrored’ incarnation and the ‘non-mirrored’ order. This hypothesis implies that the willingness of RPSC is a compromise between the ideal and reality, and rural populations need to consider their own adaptability and urban acceptance when settling in cities.

## Population and methods

### Ethics statement

The study was approved by the Research Ethics Committee of Shandong University of Finance and Economics, China (authorization number: No. 0001; 10 Aug 2018). The Research Ethics Committee of Shandong University of Finance and Economics approved the use of oral consent. As there was no benefit or physical harm in participating in the study, the respondents expressed their informed consent orally. This survey object is the adult rural population entering the city, not including minors. We use smartphones and tablets to fill in the questionnaires, so the provision of oral consent was recorded on all participant questionnaires.

### Participants

According to the theoretical analysis framework, a questionnaire was designed to investigate the willingness of RPSC in Changyi. The survey was conducted in two ways. First, using stratified sampling. According to the spatial grid, five urban communities were are selected, and the survey samples were are obtained through household access. Then, choosing the place where the rural population is concentrated to carry out the investigation, such as construction sites, labor markets, factory enterprises, etc., and obtained the survey samples through random interviews. Finally, a total of 360 questionnaires were distributed.

To ensure the credibility and authenticity of the questionnaire, we first conducted a brief face-to-face interview with the respondents, explained the main purpose of the survey to them, and ensured the confidentiality of all the data obtained according to law. Second, we issued the respondents cards with the questionnaire code to monitor the received survey and prevent repetition. Finally, some respondents were selected to verify the results of the questionnaire survey through structured and semi-structured interviews. After eliminating the invalid samples caused by data distortion, 308 valid questionnaires were obtained. The effective rate of the questionnaire was 85.6%. This corresponds to a 95% confidence level and a 25% invalid sample size.

### Study area

China has fully liberalized the restrictions on the settlement of small cities, and promoting the rural population to settle in small cities is the main direction of urbanization. It is more valuable to study the rural population settled in small and medium-sized cities. Therefore, we choose Changyi as the study area. Changyi is a county-level city, which is located between 36°25′- 37°08′N and 114°48’ –115°15’ E in the eastern coastal area of China. Changyi is a typical small and medium-sized city in China, covers the total area is 1578.7 square kilometers. Changyi governs 3 sub-districts, 12 towns. In 2018, the total population was 586,700, of which 250,800 were part of the urban registered population and 335,900 were part of the rural registered population. It is one of the top 100 scientific development cities in China, and one of the top 100 small and medium-sized cities with the most investment potential in China.

In Changyi, the per capita disposable income of urban residents is 38,461 yuan and the per capita disposable income of rural residents is 20,562 yuan. The income gap attracts the rural population into downtown areas. The new rural labor force that transferred employment amounted to 7025 people who lived downtown but did not settle down. Currently, Changyi, as a pilot city of new urbanization determined by Shandong Province, has fully liberalized the restrictions on urban household registration. The main direction for promoting new urbanization is to guide rural populations to settle down. Therefore, Changyi is a typical area to study the settlement of rural populations in small and medium-sized cities.

### Models

Since the data for measuring the willingness of RPSC come from survey data, willingness is a categorical variable that can be divided into two types: willing and unwilling. We use binary Logit regression to estimate the impact of the ‘mirrored’ incarnation and the ‘non-mirrored’ order on the willingness of RPSC. The dependent variable (the willingness of RPSC) = f(the ‘mirrored’ incarnation, the ‘non−mirrored’ order). The specific model is as follows:

$$Y=Logit(p)=ln\left(\frac{p}{1-p}\right)=\beta_{0}+\sum_{n}^{m=1}\beta_{m}x_{m}$$ (1)

where p denotes the p-value of the dependent variable (the value is between 0 and 1) and x_1_, x_2_, …, x_n_ denote the independent variables. β_0_ denotes the intercept parameters, and β_1_ denotes the coefficient of x_1_.

To clarify the effect of the ‘mirrored’ incarnation and the ‘non-mirrored’ order on the willingness of RPSC, the ‘mirrored’ incarnation variables are added to Model 1 to analyze the impact of these variables on the willingness of RPSC:

$$Y=\beta_{0}+\beta_{1}mirrored$$ (2)

The ‘non-mirrored’ order variables are added to Model 2 to analyze the impact of these variables on the willingness of RPSC:

$$Y=\beta_{0}+\beta_{1}nonmirrored$$ (3)

To verify the hypothesis, the variables of the ‘mirrored’ incarnation and the ‘non-mirrored’ order are added into Model 3. In Model 3, we analyze the influence of each variable on the RPSC willingness under the joint action of the ‘mirrored’ incarnation and the ‘non-mirrored’ order and observe the changes in the credibility and rationality of the model:

$$Y=\beta_{0}+\beta_{1}mirrored+\beta_{2}nonmirrored$$ (4)

### Variable selection

Based on the purpose of the study, we chose the willingness of RPSC as the dependent variable. We used the respondents' answers to the following question to judge the willingness of RPSC: under the existing conditions, are you willing to transfer your ‘hukou’ to the city? The choice of ‘yes’ was recoded as ‘1’, and the choice of ‘no’ was recoded as ‘0’.

According to the theoretical framework and research hypothesis of this paper, the willingness of RPSC is a realistic choice under the joint action of the ‘mirrored’ incarnation and the ‘non-mirrored’ order. Under the effect of urban labor market selection and urban social insurance and public welfare, the desire of the rural population is constantly stimulated in reality. On the basis of the above analysis, this paper chooses variables from two aspects of urban labor market selection and urban social insurance and welfare policies. Finally,19 variables that may affect RPSC willingness were selected (Table 1). The variables were classified into two groups: the ‘mirrored’ incarnation variables and the ‘non-mirrored’ order variables.

**Table 1:** Profile of independent variables and their definitions.

Variables	Definitions	Scales
**The ‘mirrored’ incarnation variables**		
Gender	Gender of respondents	1-Female, 2-Male
Age	Age of respondents	Actual age
Ethnic groups	ethnic groups of respondents	1-Han, 2-Hui, 3-Manchu, 4-Mongolian, 5-others
Educational attainment	Educational attainment of respondents	1-Did not attend school, 2-Primary school, 3- Junior high school, 4-Vocational high school/Technical secondary school, 5-High school, 6-College/Vocational college, 7-Bachelor’s degree and above
Political status	Political status of respondents	1-Communist, 2-Democratic party, 3-League member, 4-Masses
Work experience	The number of jobs respondents have worked	Calculated according to actual amount
Vocational skills	Whether the respondents have participated in vocational skills training	1-No, do not want to participate, 2- No, but willing to participate if conditions permit, 3-Participated in free training organized by the government or public welfare organizations, 4-Participated in free training organized by the work unit, 5-Participated in paid training
Income	Monthly income of respondents	Ln(Monthly income)
Household size	The number of people in a family	Calculated by actual quantity (persons)
Quantity of children	The number of children	Calculated by actual number of children (persons)
Social intercourse	Among close friends, the proportion of urban residents	1-No, 2-A small part, 3-Half, 4-Most, 5-All
Employment industry	The industry in which the respondents are currently working	1-Agriculture, forestry, animal husbandry, sideline fishery, 2-Construction industry, 3-Manufacturing industry, 4-Transportation, storage, post and telecommunications industry, 5-Wholesale and retail trade, catering industry, 6-Social service industry, 7-Education, culture and art, radio and television industry, 8-Real estate industry, 9-Finance, insurance industry, 10-State organs, party and government organs, social organizations, 11-Medical and health, sports industry, 12-Geological exploration industry, water conservancy management industry, 13-Mining industry, 14-Power, coal, water production and supply industry, 15-Scientific research and integrated technical service industry, 16-Army, 17-Others
**The ‘non-mirrored’ order variables**		
Healthcare services policy	Respondents' satisfaction with healthcare services policy	1-Satisfied, 2-Relatively satisfied, 3-Not so satisfied, 4-Dissatisfied
Children's enrollment policy	Respondents' satisfaction with their children's enrollment policy	1-Satisfied, 2-Relatively satisfied, 3-Not so satisfied, 4-Dissatisfied
Labor contract policy	Respondents' satisfaction with labor contract policy	1-Satisfied, 2-Relatively satisfied, 3-Not so satisfied, 4-Dissatisfied
Public housing policy	Respondents' satisfaction with public housing policy	1-Satisfied, 2-Relatively satisfied, 3-Not so satisfied, 4-Dissatisfied
Unemployment insurance gap	The gap between respondents and urban residents in unemployment insurance.	1-Very large, 2-Relatively large, 3-Not too large, 4-No difference, 5-Better than urban residents
Community voting rights gap	The gap between respondents and urban residents in community voting rights.	1-Very large, 2-Relatively large, 3-Not too big, 4-No difference, 5-Better than urban residents
Community safety gap	The gap between respondents and urban residents in community safety	1-Very large, 2-Relatively large, 3-Not too big, 4-No difference, 5-Better than urban residents

The first group is the ‘mirrored’ incarnation variables. The ‘mirrored’ incarnation represents the general characteristics of the rural population selected by the urban labor market. Referring to the results of existing studies, we screened the variables that represent the individual characteristics of rural populations, such as gender, age, nation, educational attainment, political status, work experience, vocational skills, income, household size, number of children, social intercourse and employment industry.

The second group is the ‘non-mirrored’ order variables, which correspond to urban public policies and rules, including healthcare services policy, children’s enrollment policy, labor contract policy, public housing policy, the unemployment insurance gap, the community voting rights gap and the community safety gap. The external institutional environment determined by urban policies and rules causes a gap between urban residents and rural residents' living standards that affects all aspects of the life of the rural population entering the city. We investigate their satisfaction with the relevant policies and their perception of the gap to determine the impact of the policies on the willingness of RPSC. By examining the relationship between these variables and the willingness of RPSC, we analyze the effect of the factors of the ‘mirrored’ incarnation and the ‘non-mirrored’ order on the willingness of RPSC.

## Results and findings

### Descriptive analysis

A descriptive analysis is given in Table 2. According to the survey data, in Changyi, only 19% of the rural population living in the downtown area is willing to settle down. Among the samples, the proportion of men and women is balanced. The majority of the rural population surveyed is of Han nationality, with minorities of Hui nationality. Most of them are young and middle-aged, with an average age of 35.96 years. Their education level is relatively low, generally below high school education. The average family size is three. Most of them have not joined any political party. Since entering the city, each rural respondent changed jobs twice on average, and many respondents admitted that they had not yet mastered good vocational skills. The average monthly income of the rural population is 3000 yuan, and most of the rural population is not in good economic condition in the city. Their social intercourse is relatively closed, and they do not have frequent contact with urban residents. The rural population is mainly engaged in manufacturing, wholesale and retail trade, and catering.

**Table 2:** Descriptive analysis.

Variables	Min	Max	Mean	SD
**the ‘mirrored’ incarnation**				
Gender	1	2	1.51	0.50
Age	22	70	35.96	9.71
Ethnic groups	1	5	1.06	0.47
Educational attainment	1	7	4.75	1.65
Political status	1	4	3.58	0.97
Work experience	1	12	2.04	1.47
Vocational skills	1	5	3.21	1.15
Income	4.83	10.28	7.80	0.72
Household size	1	8	3.88	1.28
Number of children	0	6	1.23	0.66
Social intercourse	1	5	2.69	0.91
Employment industry	2	17	4.14	2.65
**the ‘non-mirrored’ order**				
Healthcare services policy	1	5	3.54	1.52
Children’s enrollment policy	1	5	3.22	1.49
Labor contract policy	1	5	3.00	1.45
Public housing policy	1	5	3.49	1.49
Unemployment insurance gap	1	5	2.65	0.94
Community voting rights gap	1	5	2.64	0.96
Community safety gap	1	5	2.74	1.04

Regarding the living environment determined by urban policies and rules, there is indeed a gap in the living standards of urban residents and the rural population entering the city. However, the gap is not very large, especially in terms of healthcare services policy, children’s enrollment policy, labor contract policy, and public housing policy.

### Results of binary Logit regression

Based on the binary Logit regression model, we conducted regression analysis with the survey data. Models 1, 2, and 3 separately explore the influence of the ‘mirrored’ incarnation variables and the ‘non-mirrored’ order variables as well as the influence of two kinds of variables on the willingness of RPSC (see Table 3).

**Table 3:** Binary Logit regression results.

Independent variables	Model 1		Model 2		Model 3	
	B	SE	B	SE	B	SE
**The ‘mirrored’ incarnation variables**						
Gender	-0.099	0.306			-0.078	0.327
Age	0.056***	0.020			0.055**	0.022
Ethnic groups	0.627*	0.333			0.820**	0.361
Educational attainment	0.202*	0.110			0.256**	0.120
Political status	0.107	0.150			0.196	0.159
Work experience	-0.091	0.102			-0.050	0.106
Vocational skills	-0.063	0.136			-0.119	0.144
Income	-0.123	0.223			-0.064	0.238
Household size	0.035	0.124			0.040	0.132
Number of children	-0.307	0.288			-0.345	0.315
Social intercourse	0.684***	0.174			0.660***	0.185
Employment industry	0.017	0.057			0.043	0.062
**The ‘non-mirrored’ order variables**						
Healthcare services policy			-0.267**	0.134	-0.354**	0.157
Children’s enrollment policy			-0.117	0.118	-0.091	0.138
Labor contract policy			-0.088	0.129	-0.028	0.157
Public housing policy			0.346**	0.147	0.396**	0.166
Unemployment insurance gap			0.340**	0.197	0.519**	0.232
Community voting rights gap			0.154	0.181	0.135	0.212
Community safety gap			-0.464***	0.170	-0.567***	0.204
Constant	-5.463**	2.408	-0.808	0.631	-6.593**	2.669
Number of cases	301		308		301	
Pseudo R2	0.1000		0.0490		0.1507	
-2 Log likelihood	304.179		331.501		287.041	
LR chi2	33.8		17.80		50.93	

Note

*p < 0.1

**p < 0.05

***p < 0.01 in Model 1 and Model 3.

In order to clarify the influence of the ‘mirrored’ incarnation and the ‘non-mirrored’ order on the willingness of RPSC, the ‘mirrored’ incarnation variables were added in model 1, the ‘non-mirrored’ order variables were added in model 2, and then, the ‘mirrored’ incarnation and the ‘non-mirrored’ order variables were added in model 3 to analyze the influence of these variables on the willingness of RPSC. The prediction accuracy of the three models is shown in Table 4.

**Table 4:** Correctly classified.

Model 1	Model 2	Model 3
77.74%	74.35%	78.74%

#### The influence of the ‘mirrored’ incarnation variables

The ‘mirrored’ incarnation variables are included in Model 1. The pseudo R2 coefficient is 0.1 (Table 3), and the prediction accuracy of the model is 77.74% (Table 4). The results have a direct interpretation in terms of the willingness of RPSC. Age, ethnic groups, educational attainment and social intercourse passed the significance test, which is consistent with existing studies, such as those by Mohabir, Jiang, and Ma [34] and Korinek, Entwisle, and Jampaklay [35].

Age is significant at the level of 0.01 and is positively correlated with the willingness of RPSC. With the development of urbanization, the urban labor market has absorbed an increasing rural population, among which young and middle-aged rural people have more advantages. They are more likely to find jobs and settle down in cities.

When other variables are controlled, ethnic groups is strongly positively correlated with the willingness of RPSC. This may be related to the national tradition and local culture of Changyi. In the sample, the minority population is predominantly Hui people. Hui people have a strong sense of business; therefore, most rural Hui people who go to cities are likely to live together, which leads to them more willing to settle down in cities.

Educational attainment is also positive and is significant at the level of 0.05. The higher the educational attainment of the rural population is, the stronger their willingness to settle in cities. Higher educational attainment of the rural population makes it easier to find a job. On the other hand, the educational attainment of rural populations largely reflects their learning ability. People with good education have stronger learning ability and are more likely to obtain stable employment opportunities in fierce urban competition.

Social intercourse has a significant effect on the willingness of RPSC. RPSC willingness increases as the rural population makes more friends with urban residents. Good interpersonal relationships provide spiritual or material help for rural populations, which relieve their survival pressure. If they do not settle down in their current city, they may lose some of their social relations. The loss of migration is high [36–38].

Gender, political status, work experience, vocational skills, and employment industry have an impact on RPSC willingness, but not significantly. This finding shows that the differences between men and women in the urban labor market are shrinking. The rural population’s lack of work experience and good vocational skills is a common phenomenon. Disadvantages in political status and employment in different industries are not obvious weaknesses for them to participate in employment competition in cities and do not have a great impact on their willingness to settle down.

Income has no significant effect on the willingness of RPSC. This finding conflicts with the conclusion of Li, Duan, and Bai [39] but is consistent with the study of Tao, Wong, and Hui [40]. There are different opinions on the impact of income internationally [41]. With regard to the topic of this study, unlike entering the city, the rural population settles in the city not only for higher income but also for brighter personal development prospects. Therefore, the variable is not significant, which is in line with reality.

Family factors such as household size and number of children are also not significant. Due to China's family planning policy, most families are small and have only 1–3 children, which are not necessarily related to whether the rural population decides to settle in cities.

#### The influence of the ‘non-mirrored’ order variables

The ‘non-mirrored’ order variables are added in Model 2. The coefficient of pseudo R2 is 0.049 (Table 3), and the prediction accuracy of the whole model for the willingness of RPSC reaches 74.35% (Table 4). The community safety gap is significant at the level of 0.01, while the healthcare services policy, public housing policy and employment insurance gap are significant at the level of 0.05.

According to the results, public housing policy has a positive effect on RPSC willingness. In China, the public housing policy mainly refers to the housing public accumulation fund system. Urban residents must pay a portion of their income into the Public Housing Fund to be eligible for low-interest loans, which help them reduce the pressure to buy urban housing. The rural population has no access to such benefits. Housing is one of the basic conditions for rural populations to settle in cities and enhances their sense of belonging and identity. It is necessary to improve and perfect the system for providing public accumulative housing funds for rural populations who enter cities to increase their willingness to settle.

Narrowing the gap in unemployment insurance also has a positive effect on RPSC willingness. Most of the rural population entering the city is engaged in informal employment. They are often on the edge of employment and unemployment. Employment insurance is an important guarantee for them and plays a positive role in promoting their settlement.

Healthcare services policy and the decrease in the community safety gap have a negative impact on RPSC willingness. China's Rural Revitalization Strategy has greatly improved the medical conditions and living environment of rural populations, and the gap between rural and urban areas is decreasing. If the gap between urban and rural areas is large, the willingness of RPSC will be significantly increased, but a small gap will reduce the willingness, which conforms to reality.

Children’s enrollment policy, labor contract policy and the community voting rights gap have no significant impact on the willingness of RPSC. Zhang [42] noted that only a small number of farmers are willing to settle in the city for the education and enrollment of their children. Employment contract law stipulates that employment contracts are compulsory, so there is no close relationship between labor contract policy and the willingness of RPSC. Community voting rights are also protected by law, so they have no significant impact on the willingness of RPSC.

Finally, the ‘mirrored’ incarnation and the ‘non-mirrored’ order variables are all introduced into Model 3. The accuracy of the whole model is increased to 78.74% (see Table 4). Compared with Model 1 and Model 2, the rationality and credibility of the whole model are further improved. The pseudo R2 coefficient is 0.1507 (Table 3). The results reveal that the ‘mirrored’ incarnation variables that correlate with urban labor market selection have a greater impact on the willingness of RPSC. The ‘non-mirrored’ order variables corresponding to urban public policies and rules are also an important force influencing willingness.

Compared with Model 1, Model 2 and Model 3, we can see that the number of significant variables does not change. Moreover, Model 3 shows that after adding ‘non-mirrored’ order variables into the ‘mirrored’ incarnation model (Model 1), the degree of influence of each variable is significantly improved, and the accuracy and rationality of the whole model are optimized [43, 44]. This shows that there is an interaction mechanism between ‘mirrored’ factors and ‘non-mirrored’ order variables, and the combination of the two can better explain the rural population's willingness to settle down. This finding effectively verifies the hypothesis proposed in this paper: the willingness of RPSC is a realistic choice under the joint action of ‘mirrored’ incarnation and ‘non-mirrored’ order.

## Conclusion and discussion

The original value of this study lies in the use of the principle of Lacanian psychoanalytic analysis to build a framework for identifying the key influencing factors that affect RPSC willingness in small and medium-sized cities. This study makes contributions in the following aspects.

1. Introducing Lacanian psychoanalytic theory into the study, the analysis framework is constructed from the perspective of the ‘mirrored’ incarnation and the ‘non-mirrored’ order, which can effectively explain the phenomenon of RPSC, increasing the understanding of individual willingness and behavioral choice in the context of a specific social background.
2. The empirical results show that the willingness of RPSC reflects the joint action of the ‘mirrored’ incarnation and the ‘non-mirrored’ order. The rural population needs to consider their own adaptability and the urban institutional environment when settling in cities. In small and medium-sized cities, compared with the ‘non-mirrored’ order, the ‘mirrored’ incarnation has a more significant impact on RPSC.
3. Using psychological methods to study rural populations’ willingness, it provides a factual basis for local government to formulate the development strategy of promoting ‘citizenization’ and deepening urbanization in small and medium-sized cities. This can improve grassroots social governance and provide a reference for urbanization in other developing countries.

The results of this study explain the reasons why the rural population is faced with the dilemma of ‘staying in the city’ or ‘going home’. When settling in the city, the rural population needs to consider their adaptability and urban acceptance. The employment of the rural population in the city is a precondition of settlement, and urban policies and rules play a significant role in promoting the willingness of RPSC. Among the influencing factors, ‘age’, ‘ethnic groups’, ‘educational attainment’, and ‘social intercourse’, representing the ‘mirrored’ incarnation, and ‘communities’ safety gap’, ‘healthcare services policy’, ‘public housing policy’ and ‘employment insurance gap’, representing the ‘non-mirrored’ order, are more significant in affecting RPSC willingness.

According to the expression of the willingness of RPSC, we suggest strengthening employment support for rural populations in small and medium-sized cities, such as improving the education and training of rural populations and expanding social communication between rural populations and urban residents so that rural populations can meet the needs of the urban labor market. At the same time, there is still much room for improvement compared with the high demand of the rural population for urban social welfare and social security [45, 46]. Currently, the government has gradually decoupled social welfare and social security from household registration, enhancing the management system of urban-rural integration in the fields of healthcare services policy, children’s enrollment policy, and labor contract policy. In the future, it is necessary to further liberalize public housing policy, the unemployment insurance gap.

It has been proven that Lacanian psychoanalytic theory has universal applicability and extremely high application value, which can increase the understanding of individual willingness in the context of specific productivity levels and specific urban policies, build a bridge between the subjective ideas and objective policy-making.

This study analyzes the rational behavior logic of RPSC based on Lacanian psychoanalytic theory, indicates the analytical perspective of psychoanalysis on interpreting the objective world is a direction worthy of further study. It should be noted that the data used in this study are based on the data of a single city, and the conclusions have certain application limitations. In the future, more case data of small and medium-sized cities will be added to expand the applicability of the research conclusions.

## Supporting information

S1 Dataset (XLSX)
